# Wearable cardioverter defibrillator causing wound dehiscence after subcutaneous implantable cardioverter defibrillator removal

**DOI:** 10.1093/ehjcr/ytae683

**Published:** 2024-12-23

**Authors:** Tsukasa Oshima, Kenichiro Yamagata, Katsuhito Fujiu

**Affiliations:** Department of Cardiovascular Medicine, the University of Tokyo Graduate School of Medicine, 7-3-1- Hongo, Bunkyo, Tokyo 113-8655, Japan; Department of Cardiovascular Medicine, the University of Tokyo Graduate School of Medicine, 7-3-1- Hongo, Bunkyo, Tokyo 113-8655, Japan; Department of Cardiovascular Medicine, the University of Tokyo Graduate School of Medicine, 7-3-1- Hongo, Bunkyo, Tokyo 113-8655, Japan

## Case description

**Figure 1:**
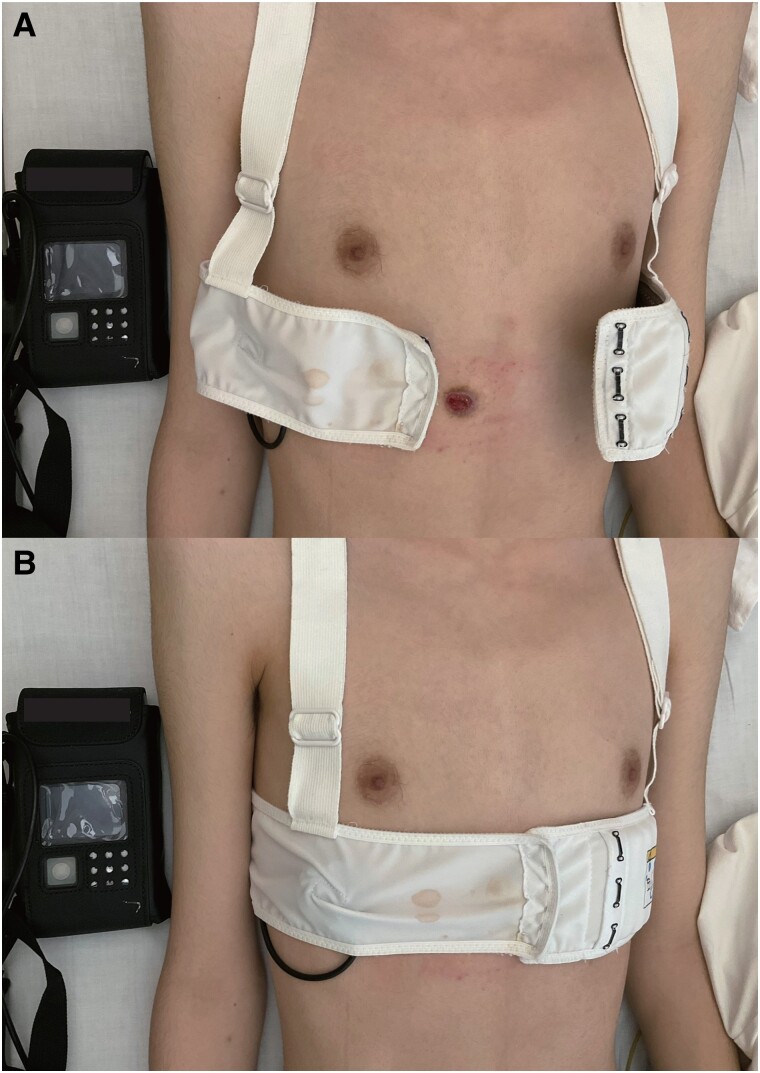
(*A*) The picture of wearable cardioverter defibrillator on and off. (*B*) The belt of the vest was just on the wound.

An 18-year-old male with arrhythmogenic right ventricular cardiomyopathy was implanted a subcutaneous implantable cardioverter defibrillator (S-ICD) 3 years ago because of concerns about future lead failures with the transvenous system. Inappropriate shocks due to T-wave oversensing occurred several times and could not be avoided by changing the settings. Therefore, replacing the S-ICD with the transvenous ICD (TV-ICD) system was planned. The S-ICD was removed first, and the TV-ICD implantation was scheduled 1 month later due to the patient’s personal reason. Meanwhile, wearable cardioverter defibrillator (WCD) was used to prevent sudden cardiac death. Although the incision wound around the xiphoid was clear 1 week after the S-ICD extraction, wound dehiscence was observed 3 weeks later (*Figure 1A*). The wound was just below the WCD chest band (*Figure 1B*). The fitting algorithm showed that the WCD size was suitable for the patient and a bigger size was too loose to detect his electrocardiogram properly. We speculated that the patient had tightened the shoulder strap more than we had instructed, causing the chest band to be pulled up and causing friction from the chest band to the wound, resulting in the wound dehiscence. The patient had no other co-morbidities and was not taking any medications that would interfere with wound healing. Transvenous ICD was successfully implanted after the wound was totally healed by local antibiotics, gentamicin sulphate to cover epidermal resident bacteria, with Vaseline and by WCD cessation.

In the event of an inappropriate shock or device infection, extraction of the S-ICD is sometimes required. We think that the progression of the underlying disease caused the ECG alteration, and it resulted in the T-wave oversensing and inappropriate shock.

Wearable cardioverter defibrillator is a temporary device used to prevent sudden cardiac death and recommended to bridge to ICD reimplantation, as in this case.^1^ However, we should notice that the vest can be just above the xiphoid wound and the tight size of the WCD could cause wound dehiscence due to its constant friction. To avoid dehiscence, we should pay attention to the size of the vest, and careful observation and gauze protection could reduce the damage on the wound.

## Data Availability

The data underlying this article is available in the article.
